# Improving extracellular vesicles visualization: From static to motion

**DOI:** 10.1038/s41598-020-62920-0

**Published:** 2020-04-16

**Authors:** Pablo Reclusa, Peter Verstraelen, Simona Taverna, Muthukumar Gunasekaran, Marzia Pucci, Isabel Pintelon, Nathalie Claes, Diego de Miguel-Pérez, Riccardo Alessandro, Sara Bals, Sunjay Kaushal, Christian Rolfo

**Affiliations:** 1 Center for Oncological Research (CORE), University of Antwerp, Antwerp, Belgium; 2 Laboratory of Cell Biology and Histology, Department of Veterinary Sciences, University of Antwerp, Antwerp, Belgium; 3 Institute of Biomedicine and Molecular Immunology (IBIM), National Research Council, Palermo, Italy; 4 Division of Cardiac Surgery, University of Maryland School of Medicine, Baltimore, MD USA; 5 Department of Biomedicine, Neuroscience and Advanced Diagnostics-Section of Biology and Genetics, University of Palermo, Palermo, Italy; 6 Electron Microscopy for Materials Science (EMAT), University of Antwerp, Antwerp, Belgium; 7 Marlene and Stewart Greenebaum Comprehensive Cancer Center, Experimental Therapeutics Research Program, University of Maryland School of Medicine, Baltimore, MD USA

**Keywords:** Fluorescence imaging, Isolation, separation and purification, Multivesicular bodies, Nucleic acids, Transporters

## Abstract

In the last decade, extracellular vesicles (EVs) have become a hot topic. The findings on EVs content and effects have made them a major field of interest in cancer research. EVs, are able to be internalized through integrins expressed in parental cells, in a tissue specific manner, as a key step of cancer progression and pre-metastatic niche formation. However, this specificity might lead to new opportunities in cancer treatment by using EVs as devices for drug delivery. For future applications of EVs in cancer, improved protocols and methods for EVs isolation and visualization are required. Our group has put efforts on developing a protocol able to track the EVs for *in vivo* internalization analysis. We showed, for the first time, the videos of labeled EVs uptake by living lung cancer cells.

## Introduction

Extracellular vesicles (EVs) were observed, for the first time, 50 years ago in plasma by Wolf, who mentioned them as “platelet dust”^1^. Two main types of EVs have been described based on their size and release mechanism: microvesicles and exosomes. Microvesicles are released, by living cells, directly from plasma membrane. Exosomes are released in extracellular space after fusion of the multivesicular bodies (MVBs) with plasma membrane^2,3^. EVs membrane consist of a lipid bilayer containing enriched sphingomyelin and decreased phosphatidylcholine^4^. In the 1980s, the Stahl’s and Johnstone’s revolutionary studies showed that nanovesicles were released by reticulocytes carrying transferrin receptors^2,3^. This process was considered a system to eliminate cellular waste and after several years, the researchers changed the concept of EVs from “cellular garbage collectors” to information shuttle. Nowadays, EVs are emerging as armed vehicles for intercellular communication^5^. EVs composition is heterogeneous and in a dynamic state, mirroring the parental cells and its pathological and physiological state^6^. Proteomic analyses revealed that EVs carry endosomal, plasma, cytosolic, and nuclear proteins. EV-cargo consists in proteins associated with membrane fusion and transport, including annexins and Rab GTPases, as well as proteins involved in EVs biogenesis and other enzymes^7,8^. They also contain nucleic acids such as mRNAs, long non-coding RNAs, microRNAs^9,10^, circular-RNAs^11^, and double-stranded DNAs^12–14^. A growing number of papers^15,16^ report that tumour-EVs play a key role in tumorigenesis, tumour growth, angiogenesis, metastasis, cancer immune escape, and drug resistance by establishing intercellular communication between cancer cells, endothelial, stromal cells, and cancer-associated fibroblasts^17–19^. Both direct and indirect evidence suggest that EVs are internalized into recipient cells, transferring functional molecules from donor to target cells^19,20^. Moreover, the stability of EVs avoids the degradation of shuttled nucleic acids; the studies on EVs are intensive in cancer research for horizontal transfer of genetic exchanges. The first papers on EV internalization proposed an interaction with recipient cells through receptor-ligand binding^21^, direct fusion with the plasma membrane^22,23^, or phagocytosis^24^. Other models reported the mechanism of EVs uptake via energy-dependent, receptor-mediated endocytosis^25,26^, or micropinocytosis^26–28^. Heusermann *et al*. reported that EVs enter into the cells as intact vesicles surfing on filopodia to sort into endocytic hot spots, then traffic within endosomes, and are finally targeted to the endoplasmatic reticulum^29^. It was also demonstrated that cellular uptake of EVs is mediated by clathrin-independent endocytosis and micropinocytosis^30^. Recently, Schneider and colleagues showed that EVs derived from alveolar macrophages were uptaked by alveolar epithelial cells in a temperature-, time-, and dose-dependent manner. The internalization was dependent on actin polymerization and dynamin. It was neither saturable nor dependent on clathrin or receptor binding. EVs uptake was improved by extracellular proteins as albumin, but was inhibited by cigarette smoke extract, via oxidative dysfunction of actin polymerization^31^. Taken together, these data indicate that EVs internalization is not a passive process^32^ and different kind of cells can internalize EVs with a specific mechanism. Actually, EVs subcellular fate within recipient cells and their mechanisms of cargo release remain unclear. It is vague how cargo delivery arises inside cells, but it is possible that either a full fusion between the EVs and endosomal membranes or a transient ‘kiss-and-run’ fusion event might carry cargo into cytoplasm before lysosome fusion with subsequent cargo degradation^33^.

Recently, EVs are appealing in cancer treatment field, due to their abilities to shuttle regulatory molecules such as different nucleic acids, lipids, and proteins, through several biofluids including blood and delivering these cargos to target cells modulating cellular activity^34^. In this study, we aim to improve and standardize the protocols for EVs visualization through different techniques as such scanning electron microscopy (SEM) and laser scanning confocal microscopy (LSCM). Previous reports have evaluated EVs cargo and trafficking in *in vitro* and *in vivo* models including lung cancer cells^35–40^, however, not showing time-lapse images during EVs uptake. On the other hand, other studies have reported similar protocols showing videos of EVs trafficking and uptake but none in lung cancer cells^41,42^. Thus, to the best of our knowledge, we described, for the first time, a protocol to observe EV-uptake and trafficking in living lung cancer cells. Our experimental model described the internalization of EVs released by CRL-5908, a non-small cell lung cancer (NSCLC) cell line resistant to tyrosine kinase inhibitors (TKIs) of first generation, as Gefitinib and Erlotinib, by the CRL-2868 cell line sensitive to these TKIs. It was already demonstrated that NSCLC cell lines released exosomes and exosomes purified by plasma of NCSLC patients are internalized by target cells to modify their phenotype^43^. Although, in the last years, EVs have been studied as biological devices, there is still no consensus on the best method to visualize the EV-uptake by recipient cells without off-target signals^44^. Recent studies, described EV-internalization analysis by confocal microscopy with PKH26 staining^45^, reporting false-positive signals due to ultracentrifugation of the PKH26 nanoparticles. Moreover, we tested two different lipophilic dyes (PKH26 and PKH67) for EVs staining^44^.

## Results

### EVs isolation and characterization

**Figure 1:**
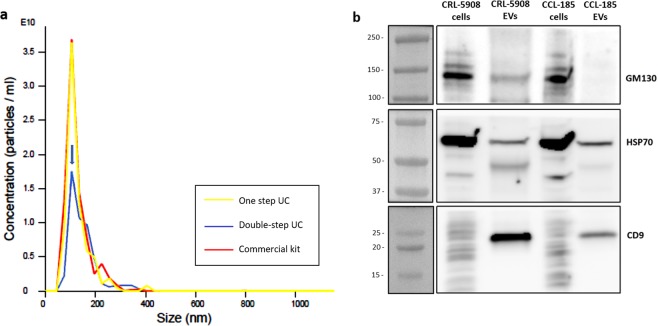
(**a**) Nanoparticle Tracking analysis (NTA) of EVs derived from CRL-5908 cells isolated with three different methods: Yellow line indicates EVs isolated with one-step ultracentrifuge method, red line EVs isolated with commercial kit, and blue line EVs isolated with double-step ultracentrifuge method (peak indicated by arrow). EVs have a mean diameter of 133.7 +/− 6.5 nm and mode of 107.5 +/− 1.7 nm. EV-concentration is expressed as numbers of particles per mL, y axis is marked from 1 to 3,5 E10. (**b**) Western-blot image of CRL-5908 and CCL-185 cells lysates and their respective isolated EVs: EVs lysates showed higher expression of CD9, lower but presence of HSP70, and absence of GM130 in comparison with cell lines lysates.

EVs were isolated from conditioned media (CM) of a CRL-5908 cell line, we compared three procedures for EVs-isolation using: commercial kit, conventional procedure based on one step of ultracentrifugation^46^, and our modified ultracentrifugation method that required a second step of ultracentrifugation (here indicated as double-step ultracentrifugation method). For this procedure, CM were collected and after centrifugation at different speeds to eliminate dead cells, cellular debris and large vesicles, were ultracentrifugated twice. This protocol, despite doubling time required and losing of vesicles, allows obtaining cleaner EV-suspensions than EVs isolated by other methods. This improvement is useful for EVs visualization with electron microscopy. Nanoparticle tracking analysis (NTA) of EVs isolated with the three different methods showed the same average size. EVs had a diameter with mean of 133.7 +/− 6.5 nm and mode of 107.5 +/− 1.7 nm (Fig. 1a). In all the experiments reported in this work, the EVs have been isolated with the double step-ultracentrifuge method, except for the experiments of comparison between the three methods of isolation described in this section.

According to the minimal requirements for EV characterization from minimal information for studies of extracellular vesicles (MISEV) 2018^46^, we identified specific EVs markers as transmembrane or GPI-anchored proteins associated to plasmatic membrane (CD9), cytosolic proteins recovered in EVs (HSP70), and transmembrane, lipid-bound and soluble proteins associated to other intracellular compartments than plasmatic membrane (GM130). The Western-blot revealed high expression of CD9, well-known marker of EVs, in CRL-5908-EVs compared to CCL-185-EVs and to whole lysates of parental cells. EVs lysates showed lower expression but presence of HSP70 and absence of GM130 in comparison with whole cell lysates (Fig. 1b).

### EVs visualization using scanning electron microscopy

**Figure 2:**
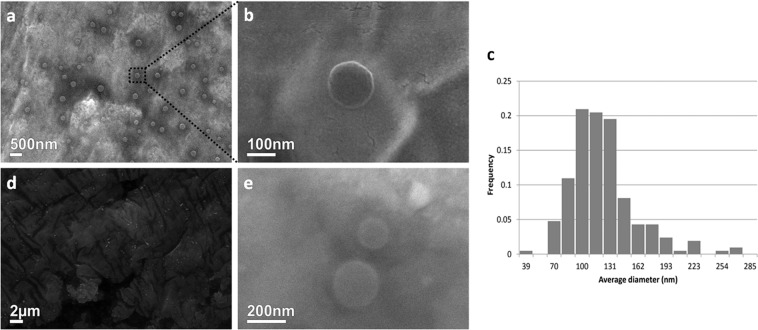
Scanning electron microscopy analysis. (**a**) EVs isolated through double ultracentrifugation. (**b**) Magnification of a single EV after double ultracentrifugation. (**c**) Histogram showing the diameter of EVs (nm) separated in its frequency with a peak on 100 to 140 nm. (**d**) Results of single ultracentrifugation at electron microscopy levels, observing protein or salt aggregates but no EVs, in comparison to double-step ultracentrifugation. (**e**) Electron microscopy analysis of EVs isolated with ExoEasy Kit revealed similar results as double-step ultracentrifugation.

In order to improve the protocol used for EVs visualization with SEM, we isolated EVs with the double-step ultracentrifugation method that allows to obtain EV-suspensions with a quite homogeneous diameter size and to eliminate protein aggregates, crystals, and other residues derived from CM. Using this protocol, vesicles with a diameter ranged between 70–190 ± 10 nm were observed (Fig. 2a,b). SEM images showed EVs with a similar diameter-range than those revealed by NTA; our data indicated that SEM analyses can be also used to accurately determine vesicles average size (Fig. 2c). Moreover, we compared the images of EVs isolated using the three different procedures discussed above. The double-step ultracentrifugation protocol (Fig. 2a,b) allows to acquire images of EVs of higher quality than the single-step ultracentrifugation, where crystal precipitates derived from CM hinder the clear identification of EVs (Fig. 2d). SEM analysis of EVs isolated with a commercial kit showed homogenous salt precipitates (Fig. 2e), however, spherical particles with typical diameter sizes already described for EVs were clearly observed (Fig. 2e).

### EVs internalization by recipient cells

**Figure 3:**
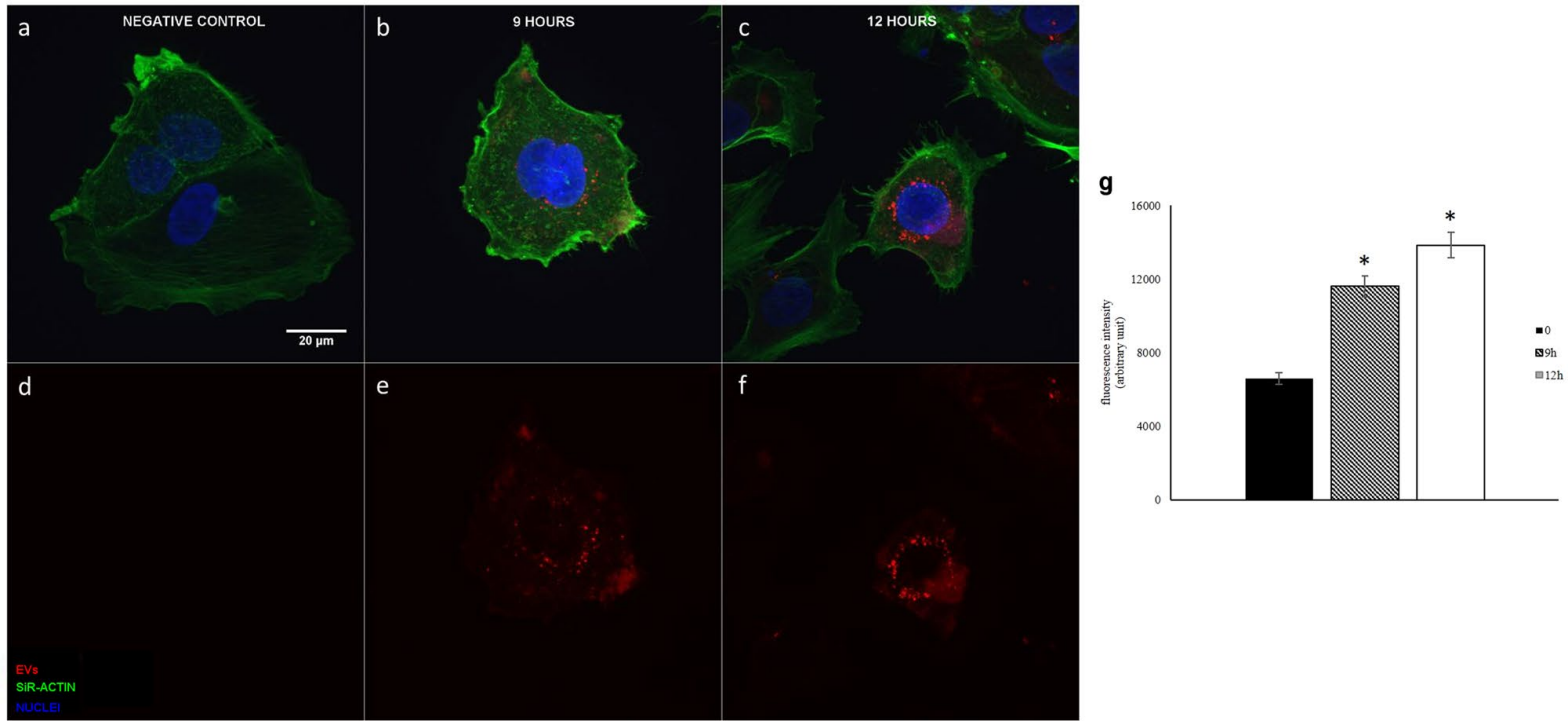
Confocal microscopy analysis of EVs internalization at different time point. Maximum intensity projections of CRL-2868 cells incubated with 20 µg/ml of CRL-5908 EVs dyed with PKH67. Negative control with no EVs (**a**,**d**), EVs internalization at 9 hours of incubation (**b**,**e**) at 12 hours (**c**,**f**). Panels a–c show merged channels, panel d–f show red channel (PKH-EVs). Semi-quantitative analysis of PKH-EVs fluorescence intensity in the cytoplasm of CRL-2868 cells, performed with ImageJ software (**g**). Values are the mean ± standard deviation of the three independent experiments. Five were measured for each experiment, *p ≤ 0.01. We acquired grayscale images that were artificially colourized, using pseudo-colours, being PKH67 dye showed in red.

**Figure 4:**
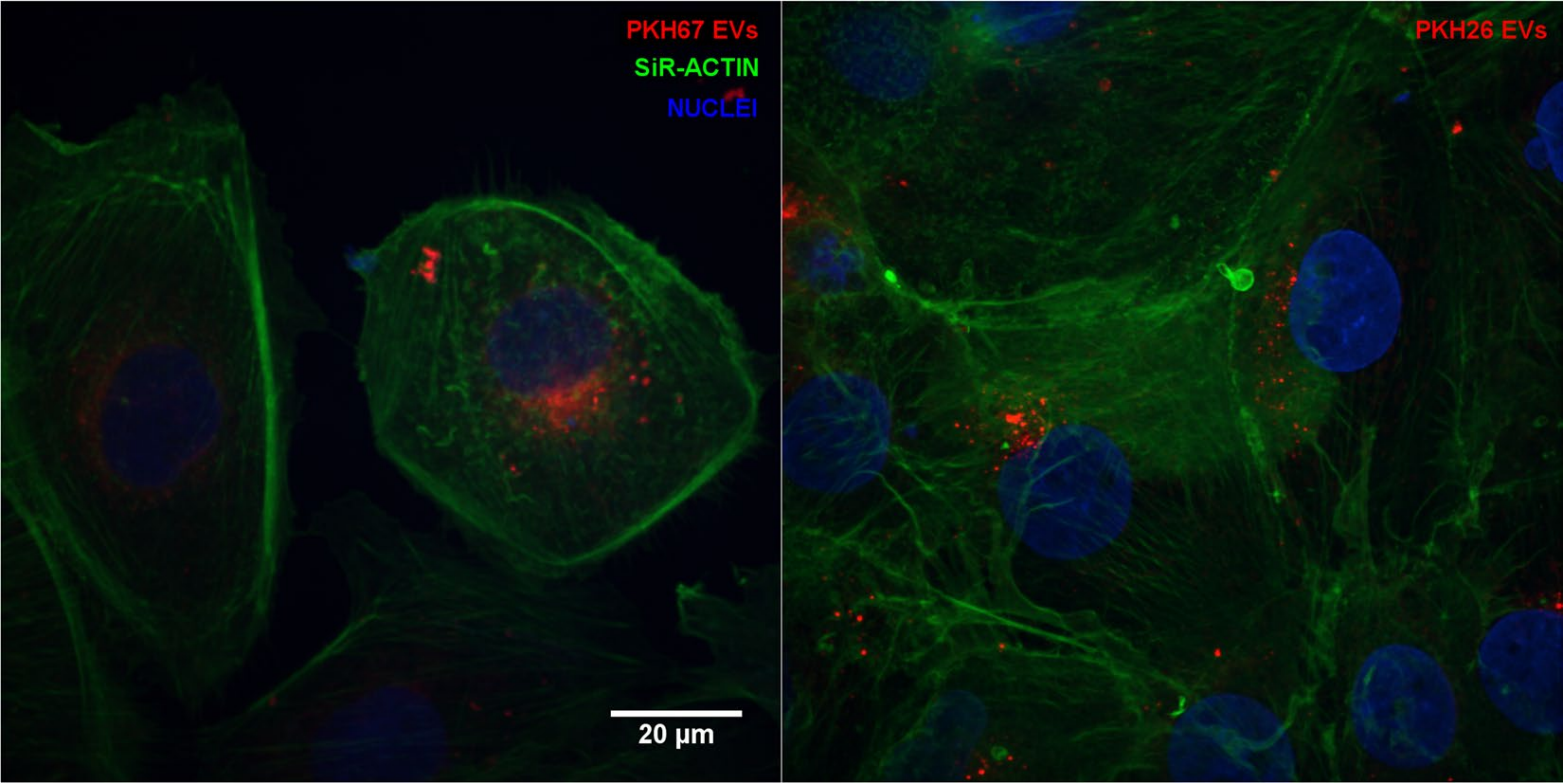
Confocal microscopy analysis of EVs stained with PKH67 and PKH26. Maximum intensity projections of CRL-2868 cells incubated with 20 μg/ml of CRL-5908 EVs dyed with PKH67 (**a**) and PKH26 (**b**) after 9 hours of incubation. We acquired grayscale images that were artificially colourized, using pseudo-colours, being both dyes showed in red.

The ability of CRL-5908-EVs to be internalized by CRL-2868 cells was investigated by examining the uptake of EVs isolated with a double step-ultracentrifuge method and labeled with the lipophilic dyes: PKH-67 a green fluorescent dye and PKH-26 a red fluorescent dye. CRL-2868 cells, treated with 20 µg/ml CRL-5908-EVs, for 9 and 12 hours, internalized the vesicles in a time-dependent manner, as showed in Fig. 3a–f. Semi-quantitative analysis of PKH-EVs fluorescence intensity in the cytoplasm of CRL-2868 cells is showed in Fig. 3g. However, it seems that PKH67 staining provided images of EVs with better intensity and contrast compared to PKH26 staining, as shown in Fig. 4.

### EVs internalization by living cells

**Figure 5:**
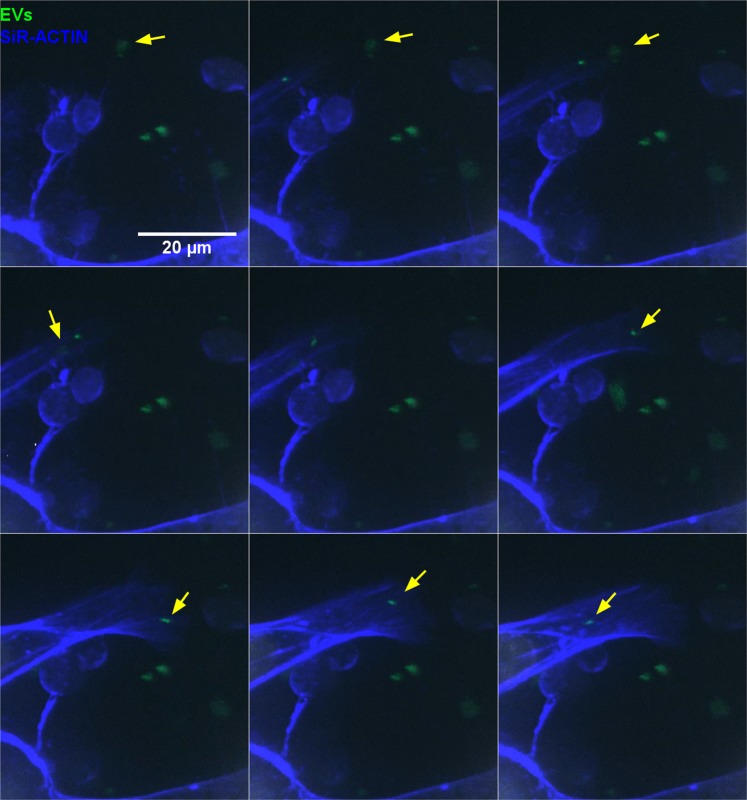
(Detailed Video 3) Confocal microscopy analysis of EVs internalization in living cells. Maximum intensity projections of CRL-2868 cells incubated with 20 μg/ml of CRL-5908-EVs dyed with PKH67. Detailed images from video 3, acquired after 6 hours of incubation, show cytoskeleton filopodia, from a cell out of the area of observation, capturing EVs. Yellow arrows point examples of uptaken EVs.

Nowadays, is evident that EVs have a key role in intercellular communication, carrying a range of molecules with a significant impact on target cells phenotype^47^. EVs are internalized by target cells; we track 20 µg/ml of labeled EVs internalization in unfixed cells, with time lapse experiments. In the images captured on isolated living cell, EVs movement is observed in the perinuclear area which becomes aggregated inside the cell in 6 hours of incubation, moreover, a prolongation of actin skeleton is observed during the first seconds of video on the bottom of cells (Video 1). As shown in Video 1, EVs seem to aggregate in the cytoplasm. In the composition of images captured on the edge of the growing cells, in Video 2, it is possible to see high concentrations of EVs in perinuclear area of the peripheral cells. More interestingly, Video 3 and Fig. 5, show EVs internalization in live cell analysis. After 6 hours of incubation, on the left area of the image, it is possible to observe cytoskeleton filopodia, from a cell out of the area of observation, capturing EVs that were free in the medium and moving them towards the cell. The restrictions of this procedure are due to the fact that laser exposition, laser power, and exposure times can alter cell morphology and cause cellular death. We show a representative video in which we optimize the condition of images acquisition.

## Discussion

It is well known that EVs are internalized by different cytotypes and uptake can depend on: EVs membrane proteins, direct membrane fusion, or endocytic processes^48^. However, the relative contribution of these mechanisms in different cell types is still unclear. Their elucidation could enhance the knowledge of EVs as biological drug carriers^26^ or implement the study of specific EVs uptake blockade to inhibit pathological process, like angiogenesis or metastasis. The isolation methods of EVs are still a bump in the road of vesicles studies, although MISEV 2018 indicates the recommended isolation methods^46^. Currently, EVs isolation gold standard is the ultracentrifugation; nevertheless, this method is time consuming and requires high technical skills. As observed in our results, when only one ultracentrifugation step is performed, the high amount of impurities and protein aggregates from CM hinders the clear identification of EVs. Thus, this protocol is not very suitable for experiments that analyze EVs morphology by electron microscopy. Our protocol with a double ultracentrifugation step, despite doubling time required for EVs isolation, provide cleaner EV-suspensions. New standardized, simple, and fast methods will be required for using EVs in clinical practice as a source of biomarkers or as drug carriers. In this field, the isolation protocol with the commercial kit based on membrane affinity could be proposed. The suspensions of vesicles obtained with this method are free of contamination of protein aggregates and of large crystal precipitates. Although useful, the commercial kit has some drawbacks regarding the use of EVs to perform functional experiments on live cells and in its implementation in clinical practice, because it could affect the downstream applications caused by its elution buffer. It is possible to eliminate the elution buffer using an ultrafiltration column, with consequent non-quantifiable losing of vesicles, that is not suitable for further applications that require a standardized amount of vesicles.

Moreover, different methods have been used for EVs staining, in our experience, PKHs are good performers for this task. PKHs use membrane labeling technology to stably incorporate a fluorescent dye with long aliphatic tails into lipid regions of the cell membrane. The labeling vehicle is an aqueous solution, isosmotic for mammalian cells without detergents or organic solvents, designed to maintain cell viability and maximizing dye solubility and staining efficiency during the labeling step. The lack of possibility to eliminate the excess of dye is one of the most important points, together with poor definition of EVs in images^49^. The use of PKH26 and PKH67 allows overcoming these problems; these dyes bind to lipid membrane of EVs, in short incubation time at room temperature. EVs, using a filtration column of 100 kDa, are able to pellet at lower speeds respect to ultracentrifugation, avoiding possible off-target staining of the target cell^44,45^. Among the two tested dyes, in our experience, PKH67 staining provided images of EVs with better intensity and contrast compared to PKH26 staining^31,47,50^.

EVs are of major interest as drug carriers due to their tissue specificity. However, in order to track the EVs, their visualization is a key step. For the first time, we have shown EVs internalization in living cell experiments and we have developed a protocol to perform it. As shown in Video 3, EVs can be internalized through filopodia extensions from the cell membranes. In the video, it is possible to see how the filopodia moves towards the EVs, capturing them into the intracellular space. In this study, we described a modified protocol for EVs isolation and the methods to visualize EVs in SEM and to track EVs internalization in living cells using confocal microscopy. Further studies need to elucidate the mechanisms of EVs internalization by target cells and the release of EV-cargo in intracellular space.

## Methods

### EVs isolation

EVs were isolated from conditioned media (CM) of CRL-5908 cell line. Moreover, EVs were also isolated from CCL-185 cell line for western-blot characterization experiments. Cells were plated in 175 cm^2^ flasks, at a concentration of 25000 cells/cm^2^, in RPMI-1640 L-Glutamine (Gibco. Ref 11875-093) supplemented with 10% FBS, 1% Pen-Strep and 1% L-Glutamine (complete medium). After 24 h of incubation, when the cells were subconfluent (≈80%), the culture medium was removed, and cells were washed with PBS. New complete medium (40 ml) with EV-depleted bovine serum was added to the cells and incubated for 24 h. Following incubation, the CM was collected and centrifuged at 500 g × 5′, 3000 g × 15′ at 4 °C to eliminate dead cells and cellular debris respectively. The CM was centrifuged at 10000 g × 30′, at 4 °C, in QuickSeal® Polypropylene tubes (Beckman Coulter, Ref. 342414) to remove large EVs (diameter about of 1000 nm). Then CM was collected and ultracentrifuged at 100000 g × 1 h 45′ in Quick-Seal UltraClear tubes (Beckman Coulter, Ref. 344326), to collect EVs. For the double step ultracentrifugation method, CM was ultracentrifuged twice at 100000 g × 1 h 45′. The different steps of ultracentrifugation are performed both with a 70ti Rotor (Beckman Coulter). The pellet of vesicles collected by each tube was resuspended in 40 µL of PBS. In the isolation protocol with commercial kit, ExoEasy Maxi Kit (Qiagen. Ref 76064), CM were centrifugated at 10000 g for 30′, in order to eliminate large vesicles (about 1000 nm of diameter), before to apply the manufacturer’s instructions of isolation based on membrane affinity spin columns. After this procedure, EVs were used immediately for the experiments or stored at −20 °C until being used.

### Nanoparticle tracking analysis

Nanoparticle tracking analysis (NTA) was performed to observe EVs size distribution and concentration using a NanoSight NS300 system (Malvern Instruments). Analysis was performed using NTA 3.1 software with threshold equal to 5 and default settings. EVs were resuspended in PBS.

### Western-blotting

CRL-5908 and CCL-185 cells were collected and washed with PBS in a 350 g × 5′ centrifugation. Previously isolated EVs and cells from both cell lines were lysed ice-cold Cell Lysis Buffer (Cell Signaling Technology. Ref 9803 S) according to manufacturer recommendations. Proteins were quantified with the Pierce™ BCA Protein Assay Kit (Thermo Fisher Scientific. Ref 23225) in Infinite® 200 PRO NanoQuant (TECAN). Thirty μg of protein from each sample were run in Mini-PROTEAN TGX 4–20% gels (Bio-Rad. Ref 561094) and transferred to nitrocellulose membranes (Bio-Rad. 162-0115). Membranes were blocked with 5% non-fat dry milk in Tris-buffered saline +1% Tween20 and incubated with primary antibodies, mouse monoclonal anti-CD9 (Thermo Fisher Scientific. Ref 10626D), mouse monoclonal anti-Hsp70 (BD Biosciences. Ref 554243), and mouse monoclonal anti-GM130 (BD Biosciences. Ref 610822), at 1:1000 dilution overnight at 4 °C. Then, membranes were washed with Tris-buffered saline +1% Tween20 and incubated with Goat anti-mouse HRP-conjugated secondary antibody (Abcam. Ref ab97023) at 1:5000 for 1 hour at room temperature. Finally, they were revealed using Clarity™ Western ECL Substrate (Bio-Rad. Ref 1705061) and pictures were taken in the ImageQuant LAS 4000 (GE HealthCare Life Sciences).

### Scanning electron microscopy

In the workflow of EVs observation by scanning electron microscopy (SEM), the selection of isolation protocol is a crucial step. The EVs isolated with three different methods described in the section EVs isolation were fixed with 2.5% glutaraldehyde in PBS for 10 min^35^. After the fixation step, EVs were attached and dried on stubs. In order to inhibit charging and to improve the secondary electron signal, a 15 nm conductive gold layer was deposited by sputter coating. The images of EVs captured by SEM were acquired using a FEI Quanta 250 FEG environmental scanning electron microscope. The microscope was operated at 30 kV.

### Confocal microscopy analysis

Sterile coverslips were placed in a 24 well plate and treated with 0.1% gelatin diluted in sterile water for 45 minutes at 37 °C. In each well, 50000 CRL-2868 cells were plated and incubated at 37 °C and 5% CO_2_ for 24 h. EVs (20 µg/ml) were incubated with PKH26 (λexc 551 nm λem 567 nm) (Sigma-Aldrich. Ref PKH26GL-1KT) and/or PKH67 (λexc 490 nm λem 502 nm) (Sigma-Aldrich Ref PKH67GL-1KT). Fluorescein filters are suitable for PKH67 detection and rhodamine or phycoerythrin filters are suitable for PKH26 detection.

The labeling of EVs with PKH67 and PKH26 was performed applying the manufacturer’s instructions. Briefly, 2× Dye Solution was prepared in Diluent C by adding 4 μL of the PKH26 ethanolic dye solution to 1 mL of Diluent C in a centrifuge tube and mix well to disperse. An EVs/dye solution (1:1 dilution EVs/dye, no less than 100 μg of EVs suspended in 100 μL of PBS) that was incubated at room temperature for 10 min and centrifuged on filter column at 14000 g for 10 min at 4 °C. EVs pellet was washed in PBS, centrifuging on filter column (0.5 ml ultrafiltration units, Amicon, Merck) to avoid free dye in the suspension and to eliminate the excess of dye in the EVs membrane. This step was repeated at least twice, until the supernatant was transparent after the centrifugation and filtration. The staining of the EVs was done maximum 3 h prior to the treatment. Nine or twelve hours after EVs treatment, CRL-2868 cells were fixed with 4% paraformaldehyde for 1 h. After permeabilization with Triton X-100 for 3 min, cells were stained with SiR-actin (100 nM, SiR-actin; λexc 640 nm λem 705 nm; Cytoskeleton Inc CY-SC001). Cells were preserved and nuclei were visualized using VECTASHIELD Antifade Mounting Medium with DAPI (Vector Laboratories. Ref H-1200). In order to obtain detailed images of intracellular EVs labelled with PKH26, confocal recordings were made using a Leica TSC SP8 scanning laser confocal system (Leica Microsystems, Wetzlar, Germany), attached to a DMI8 inverted microscope and equipped with a 405-diode laser and a white light laser. On the white light laser, excitation wavelengths were set at 551 nm and 645 nm for the excitation of EVs and SiR-actin respectively. DAPI staining was visualized by the 405-diode laser. An Acousto-Optical Beam Splitter (AOBS) was used to select the corresponding emission spectra, i.e. 410 nm – 460 nm for DAPI 560 nm – 640 nm for EVs-PKH26, 658 nm – 783 nm for SiR-actin. The signal of the EVs-PKH26 was detected using a classic PMT detector, while the DAPI and SiR-actin were visualized by a Hybrid or HyD detector in standard mode. Recordings were performed using a 63× water immersion objective (N.A. 1.2) and a pinhole of 1 Airy Unit. Images were taken with a pixel size of 1024 × 1024. Pixel saturation was prevented by adjusting the laser intensities and amplifier gains of the detectors. To avoid cross-talk between the three fluorophores image acquisition was performed sequentially. For each recording a z-stack was taken. Maximum intensity projections were obtained via the Leica TSC SP8 software. All detectors use grey levels, and thus the colours observed are pseudo-colours. High magnification images of EVs labelled with PKH67 were recorded as described in the next paragraph for live cell imaging.

### Live cell imaging

For live imaging of EVs uptake and intracellular mobility, CRL-2868 cells were grown in 96-well plates with chimney wells (Greiner µClear 655090) at a density of 2000 cells per well, in duplicates, for 24 h before EVs incubation. EVs were labeled with PKH67 as described above. Five hours before imaging, cells were incubated with 20 µg/ml of stained EVs diluted in RPMI-1640. In order to allow robust identification of nuclei and the cytoskeleton, the cultures were incubated with the membrane-permeable dyes Hoechst 33342 (final concentration 10 µg/ml, ThermoFisher H3570) and SiR-actin (final concentration 100 nM, Cytoskeleton Inc CY-SC001) respectively, for 30 min at 37 °C, 1 h before starting the image acquisition. Imaging was based on previous internalization data by our group^41,42^. Confocal imaging was performed on a dual spinning disk confocal microscope (UltraVIEW VoX system with Volocity software, PerkinElmer, Seer Green, United Kingdom) at 37 °C and 5% CO_2_ in growth medium. A water immersion objective (60×, NA 1.2, Plan Apochromat) was used to acquire images at high resolution. The motorized stage was programmed to visit multiple locations in different wells with intervals of 5 minutes per location. At each location and time point, the Zfocus was determined by means of image autofocus in the SiR-actin channel, after which 20 Zplanes were recorded with 1 µm spacing. For each channel (Hoechst λexc 405 λem 445, EVs λexc 488 λem 525, SiR-actin λexc 640 λem 705 nm), acquisition parameters were set to limit photodamage while having sufficient signal-to-noise ratio during the course of an overnight experiment. Images were exported as tiff file format for further analysis in ImageJ freeware^43^. Hyper stacks containing the time frames of each fluorescence channel were construed, after which channels were merged into RGB colour frames. The resulting image stack was exported as AVI with a frame rate of 5 frames/sec (Videos 1 and 2) and 2 frames/sec (Video 3). Videos were edited afterwards in order to focus more on the field and time frame of interest. EVs are represented in green while SiR-Actin in blue in the Videos.

### Statistical analysis

Data were expressed as mean ± standard deviation of three independent experiments. Statistical analysis was performed by using an unpaired Student’s t-test. Differences were considered to be significant when P values were smaller than 0.05.

## Supplementary information

Video 1.

Video 2.

Video 3.

Supplementary information.
