# Supplementary material for: Improving extracellular vesicles visualization: From static to motion
Source: Sci Rep. 2020 Apr 16;10:6494. doi: 10.1038/s41598-020-62920-0 (PMC7162928; doi:10.1038/s41598-020-62920-0)
Supplement: Supplementary file 4 — Supplementary information. [file 41598_2020_62920_MOESM4_ESM.pdf]

Improving extracellular vesicles visualization: From static to motion

Pablo Reclusa[1*] , Peter Verstraelen[2*] , Simona Taverna[3*] Muthukumar Gunasekaran[4], Marzia Pucci[5] , Isabel Pintelon[2] , Nathalie Claes[6] , Diego De Miguel-Pérez[7] , Riccardo Alessandro[5] , Sara Bals[6] , Sunjay Kaushal[3] & Christian Rolfo[1,3,7#]

Supplementary data

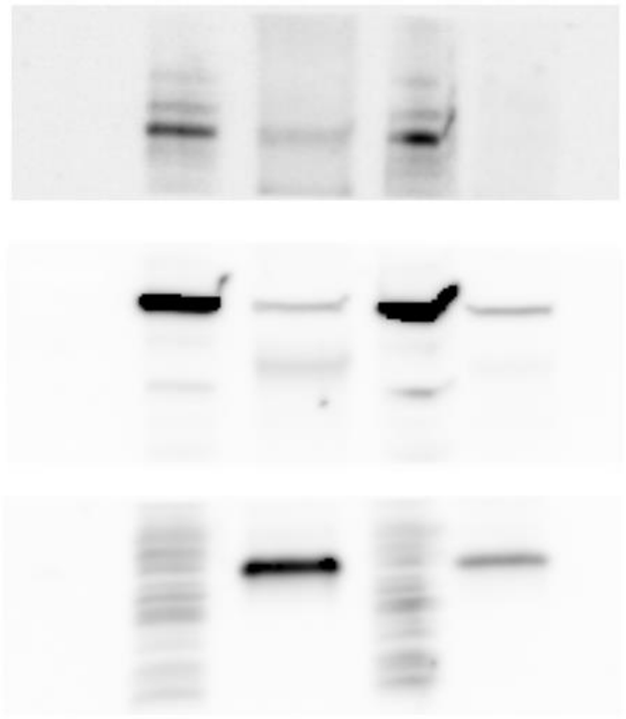

Supplementary figure S1: Original images from Western blot analysis. First well corresponded to the ladder marker not visible with chemiluminiscence. Second well corresponded to CRL-5908 cells, third to CRL-5908 EVs, fourth to CCL-185 cells and fifth to CCL-185 EVs. Blots from top to bottom are GM130, HSP70 and CD9.
